# Prevalence of gastrointestinal parasites in horses in the district of Abidjan, Ivory Coast

**DOI:** 10.1038/s41598-026-56528-z

**Published:** 2026-06-12

**Authors:** Noumouké Kante, Lanceï Kaba, Kouadio-Ba Idrissa N’Gotta, Almamy Ousmane Deen Camara, Albert Sourou Salako, Kouramoudou Berete, Karim Tuo, Assi Fiacre-Tanguy N’Docho, Éric Gondo, Marie Marlène Cyriaque Teohou, Adjoua Aicha N’Gotta, Abdoulaye Kuan Traore, Nicolas Barro

**Affiliations:** 1 Département de Médecine Vétérinaire, Institut Supérieur Des Sciences Et de Médecine Vétérinaire (ISSMV) de Dalaba, Dalaba, Guinea; 2 Laboratoire Central Vétérinaire de Diagnostic, Ministère de L’Agriculture Et de L’Elevage, Conakry, Guinea; 3 Unité de Parasitologie, Institut Pasteur de Côte d’Ivoire, Ivory Coast, Abidjan, Ivory Coast; 4 Direction Nationale Des Services Vétérinaire de Côte d’Ivoire, Abidjan, Ivory Coast; 5 Université Norbert ZONGO, Koudougou, Burkina Faso; 6 Université Joseph KI-ZERBO, Ouagadougou, Burkina Faso

**Keywords:** Horse, Coprology, Gastrointestinal parasites, Prevalence, Côte d’Ivoire, Diseases, Ecology, Ecology, Microbiology, Zoology

## Abstract

Gastrointestinal parasitic infections constitute a major constraint to equine health and productivity, particularly in tropical regions where climatic and environmental conditions favor parasite survival and transmission. In Côte d’Ivoire, epidemiological data regarding gastrointestinal parasites in horses remain limited, especially in urban and peri-urban settings such as the district of Abidjan. This study aimed to determine the prevalence, diversity, and associated risk factors of gastrointestinal parasites affecting horses in the study area. A descriptive and analytical cross-sectional study was conducted from September 2024 to February 2025 among 277 horses originating from 19 equine facilities, including 8 riding schools and 11 breeding farms, located in the district of Abidjan. Fresh fecal samples were collected and analyzed using four coprological techniques: direct examination, flotation, the simplified Ritchie concentration method, and Ziehl–Neelsen staining. Statistical analyses, including chi-square tests and logistic regression, were performed to identify factors significantly associated with parasitic infections at a significance threshold of p ≤ 0.05. The overall prevalence of gastrointestinal parasites was 88.80%, indicating widespread circulation of equine gastrointestinal parasites within the study area. Fifteen parasite species were identified, with predominance of Strongylus spp. (48.37%), Parascaris equorum (40.07%), cyathostomins (Cyathostominae) (30.68%), Fasciola hepatica (29.24%), and Cryptosporidium parvum (28.51%). Nematodes represented the predominant parasitic group. Significant associations were observed between parasitic infections and management-related factors, particularly feeding practices and type of equine activity. Horses from riding schools appeared more exposed to certain parasitic infections compared with horses raised on breeding farms. The high prevalence and diversity of gastrointestinal parasites observed among horses in the district of Abidjan highlight an important equine health concern in Côte d’Ivoire. These findings emphasize the need to strengthen integrated parasite control strategies, including regular parasitological monitoring, improved hygiene and management practices, rational use of anthelmintics to reduce the risk of resistance, and targeted deworming programs. Reinforcing veterinary surveillance and increasing awareness among horse owners and managers could substantially contribute to improving equine health and productivity in the study area.

## Introduction

Gastrointestinal parasitic infections constitute one of the major constraints affecting equine health and productivity worldwide, particularly in tropical and subtropical regions where climatic and environmental conditions favor the development, survival, and transmission of parasites. Horses infected with gastrointestinal parasites may present weight loss, anemia, digestive disorders, reduced physical performance, poor body condition, and, in severe cases, mortality, thereby causing important economic losses for breeders, owners, and equestrian facilities^1^. Among the most frequently reported gastrointestinal parasites in equids are strongyles, cyathostomins, Parascaris equorum, Strongyloides westeri, Anoplocephala spp., and several protozoan parasites, many of which are responsible for significant health and welfare problems in horses^2^.

The epidemiology of equine gastrointestinal parasites is strongly influenced by environmental and management-related factors such as climate, humidity, stocking density, pasture contamination, feeding systems, hygiene conditions, and deworming practices. In tropical environments, warm temperatures and high humidity promote the maturation and persistence of infective parasitic stages in the environment, thereby increasing the risk of transmission among animals^3^. Previous studies conducted in Ethiopia, Nigeria, Nepal, and Senegal have reported high prevalences and considerable diversity of gastrointestinal parasites in horses and other equids, emphasizing the important role of management systems and sanitary conditions in parasite dissemination^1,4–6,7,8,9^.

In Côte d’Ivoire, information regarding gastrointestinal parasites affecting horses remains limited despite the increasing use of horses for recreational, sporting, security, and breeding purposes, particularly in urban and peri-urban areas such as the district of Abidjan. The district of Abidjan is characterized by a humid equatorial climate, dense urbanization, and close animal contact, conditions that may favor the persistence and circulation of gastrointestinal parasites. However, few epidemiological studies have investigated the prevalence, diversity, and associated risk factors of equine gastrointestinal parasitoses in this area.

Understanding the epidemiological profile of gastrointestinal parasites in horses is essential for improving parasite control strategies, reducing economic losses, and enhancing equine health and welfare. Therefore, the present study aimed to determine the prevalence and diversity of gastrointestinal parasites in horses in the district of Abidjan and to identify the main management-related factors associated with parasitic infections.

## Materials and methods

### Study area and study period

The study was conducted in the district of Abidjan from 1 September 2024 to 16 February 2025. The district is located on the southern coast of Côte d’Ivoire between latitudes 5°00′ and 5°30′ North and longitudes 3°45′ and 4°15′ West. It covers an area of approximately 2,119 km^2^ and includes 13 municipalities: Abobo, Adjamé, Anyama, Attécoubé, Bingerville, Cocody, Koumassi, Marcory, Plateau, Port-Bouët, Songon, Treichville, and Yopougon.

**Fig. 1:**
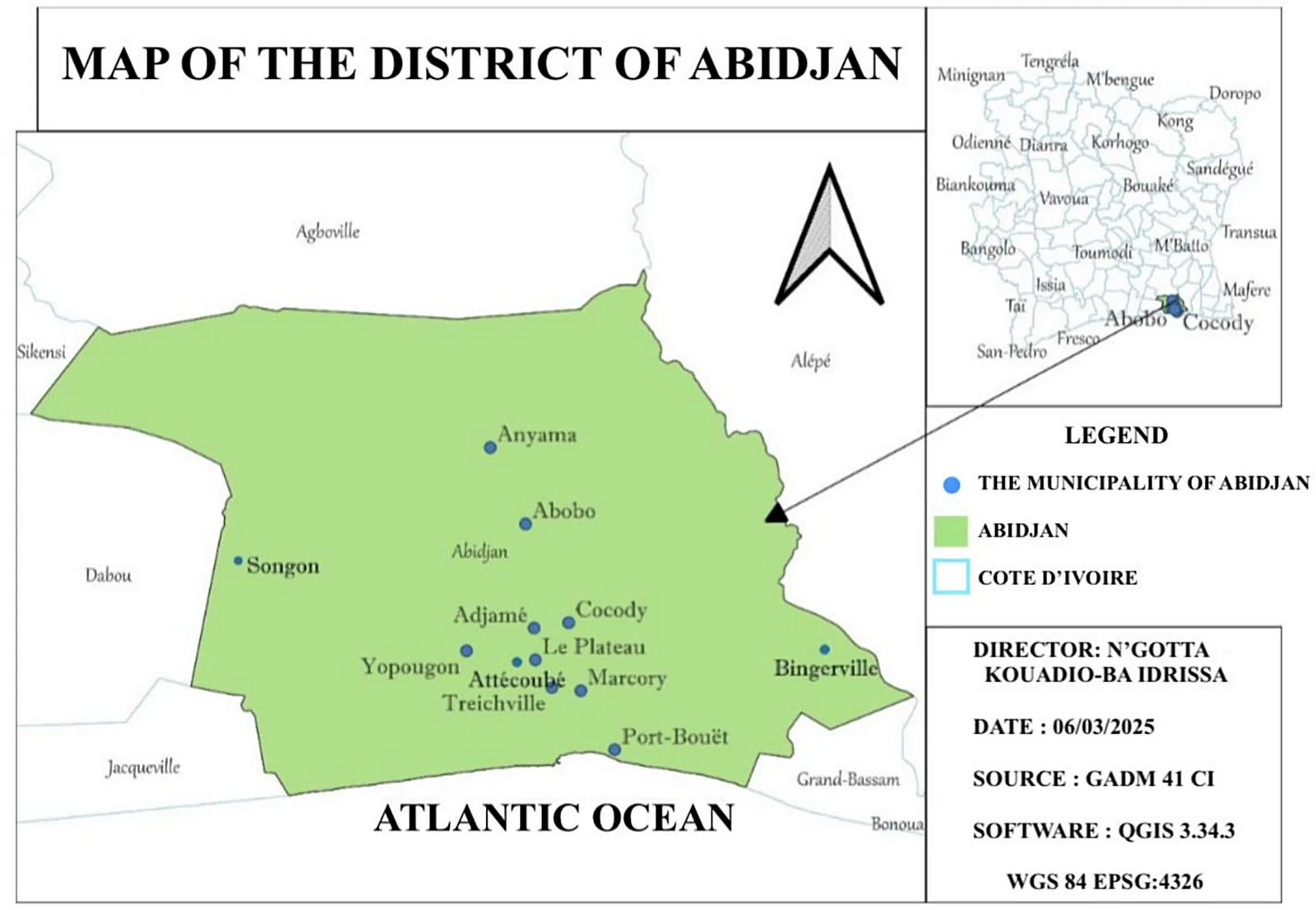
Map of the district of Abidjan.

The district of Abidjan is characterized by predominantly flat and slightly hilly relief, with an average altitude of approximately 50 m above sea level. The climate is humid equatorial, with two rainy seasons and two dry seasons annually. Temperatures generally range between 25 °C and 30 °C, while relative humidity varies from 80 to 90% because of the proximity to the Atlantic Ocean. These climatic and environmental conditions, combined with dense urbanization and close animal contact, may favor the persistence, survival, and transmission of gastrointestinal parasites among horses. (Fig. 1).

### Sampling strategy

To obtain a representative assessment of gastrointestinal parasite prevalence according to horse management systems, a stratified sampling approach was adopted based on the type of equine facility. Two categories of facilities were considered: riding schools, characterized mainly by the sporting and recreational use of horses, and breeding farms, where horses were primarily raised for breeding, work, or commercial purposes.

Following this stratification, all horses present in the selected facilities during the study period were included in the study. A total of 277 horses originating from 19 equine facilities, including 8 riding schools and 11 breeding farms, were sampled.

Additional epidemiological information, including age, sex, breed, type of equine activity, and deworming practices, was collected using structured questionnaires administered to horse owners and facility managers.

Fresh fecal samples were collected directly from the rectum of horses using sterile gloves. Each sample was labeled with a unique identification code corresponding to the horse and the facility of origin. Samples were placed in sterile containers, sealed hermetically, and transported under refrigerated conditions (+ 4 °C) to prevent deterioration before laboratory analysis. All analyses were performed within 24 h at the parasitology laboratory of the Institut Pasteur de Côte d’Ivoire.

### Coprological analyses

To improve the detection and identification of gastrointestinal parasites, fecal samples were microscopically examined using four coprological techniques: direct examination, flotation technique, simplified Ritchie concentration method, and Ziehl–Neelsen staining. These complementary methods were selected to enhance the detection of parasite eggs, larvae, oocysts, and cysts associated with equine gastrointestinal parasitoses. All laboratory procedures were performed according to the methods described by Bailenger (1956).

### Statistical analysis

Data were entered, cleaned, and processed using Microsoft Excel Pro 2021 (Microsoft Corporation, USA) and RStudio version 2024.12.1 + 563 (R Foundation for Statistical Computing, Vienna, Austria). Descriptive statistics were used to summarize the data, including the calculation of parasite prevalence, frequencies, and proportions according to horse characteristics and management-related variables. The overall prevalence of gastrointestinal parasites was determined as the proportion of infected horses among the total number of examined animals.

Associations between gastrointestinal parasitic infections and explanatory variables, including the type of structure (riding schools versus breeding farms), were initially assessed using Pearson’s chi-square test or Fisher’s exact test where appropriate, depending on expected cell counts. Variables showing potential associations with parasitic infections were subsequently included in a logistic regression model to identify factors significantly associated with the occurrence of gastrointestinal parasites in horses.

Multivariate logistic regression analysis was performed to estimate odds ratios (ORs) and their corresponding 95% confidence intervals (95% CIs). This analysis allowed the evaluation of the effect of management-related factors on the probability of parasitic infection while controlling for potential confounding variables. An odds ratio lower than 1 was interpreted as a protective effect of the considered factor against parasitic infection.

The results were considered statistically significant when the p-value was less than or equal to 0.05 (p ≤ 0.05).

## Results

The ages of the 277 horses ranged from 3 months to 27 years. The population comprised 161 males (58.12%) and 116 females (41.88%), the majority of which were local breeds (70.04%), followed by exotic breeds (25.63%) and mixed breeds (4.33%).

### Identification and frequency of parasitic species

**Fig. 2:**
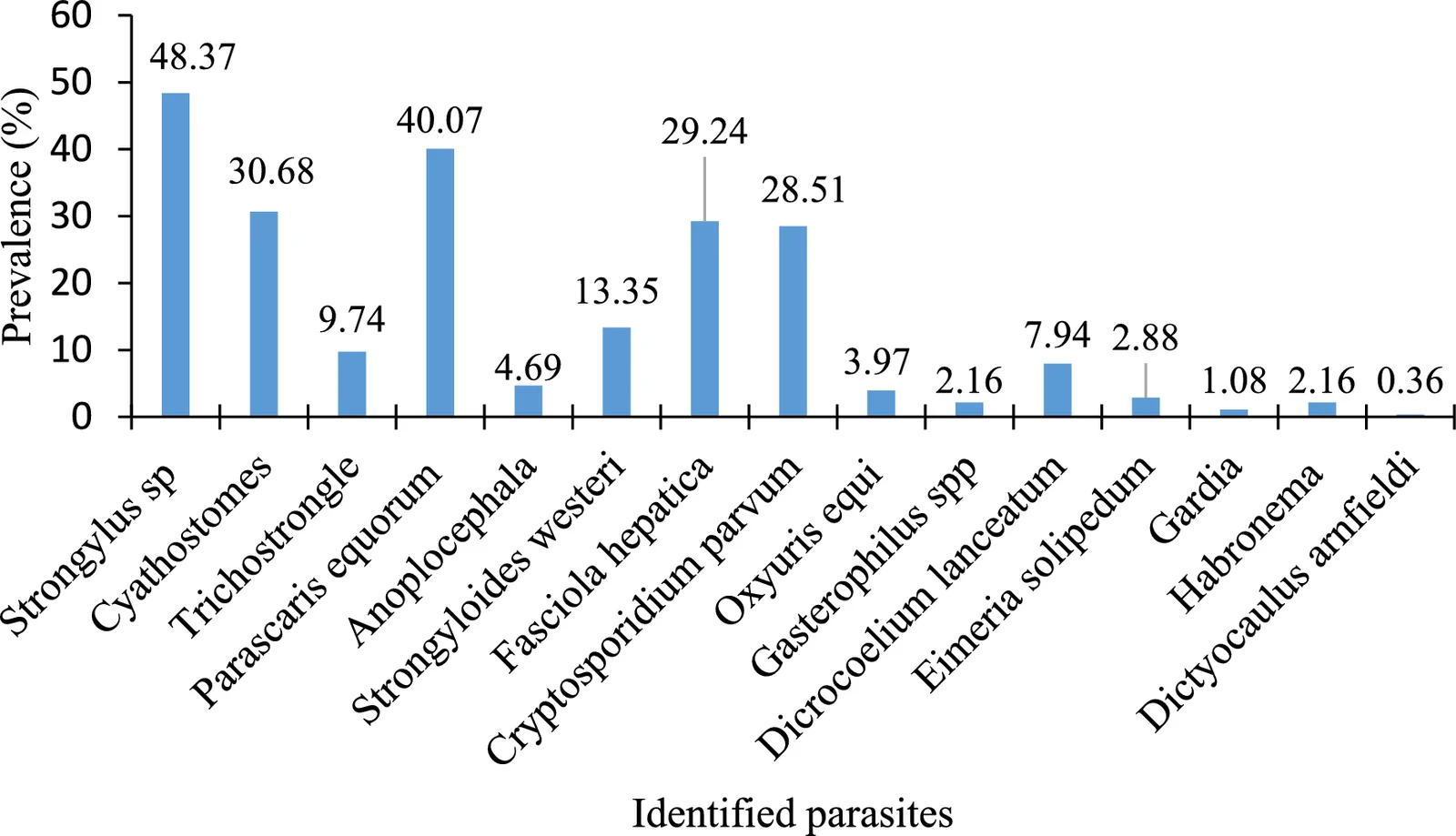
Prevalence of parasites identified in Abidjan district.

Coprological analysis of horses from riding schools and farms in the Abidjan district identified fifteen species of parasites (Fig. 2) affecting the gastrointestinal tract of equids. Nematodes, including Cyathostomes, Dictyocaulus arnfieldi, Habronema, Parascaris equorum, Strongylus sp., Strongyloides westeri, Trichostrongles and Oxyuris equi, were generally the dominant class. Next in frequency were protozoa (Cryptosporidium parvum, Eimeria solipedum, Giardia), trematodes (Fasciola hepatica, Dicrocoelium lanceatum) and, more marginally, cestodes (Anoplocephala). Overall, parasitic infestations were detected in 246 horses out of 277, for an overall rate of 88.80%, indicating a worrisome epidemiological situation. The most frequent species (Fig. 2) were strongyles, followed by Parascaris equorum, Cyathostomes, Fasciola hepatica and Cryptosporidium parvum, whereas the other species were represented at lower frequencies.

### Logistic regression analysis of factors associated with gastrointestinal parasitic infections in horses in Abidjan district

To further investigate the factors associated with gastrointestinal parasitic infections in horses, a multivariate logistic regression analysis was performed using the type of structure as an explanatory variable. This analysis aimed to evaluate whether horses raised in breeding farms or riding schools differed significantly in their exposure to specific parasitic infections. The results showed significant associations between the type of structure and the occurrence of several gastrointestinal parasites, highlighting the influence of management practices and environmental conditions on parasite transmission dynamics in the Abidjan district. The detailed results of the logistic regression analysis are presented in Table 1.

**Table 1:** Logistic regression analysis of factors associated with gastrointestinal parasitic infections in horses in Abidjan district.

variables	Categories	OR	95% CI	p-value
Type of structure	Breeding farm vs Riding school (Strongylus sp.)	0.46	0.28–0.75	0.002
Type of structure	Breeding farm vs Riding school (Trichostrongyles)	0.42	0.18–0.96	0.042
Type of structure	Breeding farm vs Riding school (Anoplocephala spp.)	0.07	0.01–0.58	0.011
Type of structure	Breeding farm vs Riding school (Fasciola hepatica)	0.04	0.01–0.22	< 0.001
Type of structure	Breeding farm vs Riding school (Cryptosporidium parvum)	0.28	0.14–0.55	< 0.001

OR: Odds Ratio; CI: Confidence Interval. An OR < 1 indicates a protective effect of breeding farms compared with riding schools.

The logistic regression analysis demonstrated that the type of structure significantly influenced the occurrence of several gastrointestinal parasites in horses. Horses raised in breeding farms were significantly less exposed to Strongylus sp., Trichostrongyles, Anoplocephala spp., Fasciola hepatica, and Cryptosporidium parvum compared with horses from riding schools. The strongest protective effect was observed for Fasciola hepatica (OR = 0.04; p < 0.001) and Anoplocephala spp. (OR = 0.07; p = 0.011). These findings suggest that management conditions in breeding farms may reduce the risk of exposure to several gastrointestinal parasites. Conversely, horses kept in riding schools appeared to be more vulnerable to parasitic infections, possibly due to higher animal density, increased movement, and differences in hygiene and deworming practices.

#### Implementation of strategic deworming programs

Regular and targeted anthelmintic treatments should be implemented based on coprological results to improve effectiveness and reduce the risk of drug resistance.

#### Strengthening parasitological surveillance

Routine fecal examinations should be promoted to monitor parasite burden and guide appropriate treatment strategies in both riding schools and breeding farms.

#### Improvement of management practices

Enhanced hygiene measures, including regular cleaning of stables and proper manure management, should be adopted. In addition, reducing animal density and improving pasture management can help limit parasite transmission.

#### Capacity building and awareness

Training and sensitization programs for horse owners and stable managers are essential to promote good husbandry practices and increase awareness of parasitic risks.

#### Enhancement of veterinary services

Strengthening veterinary support and regular health monitoring is crucial to ensure early diagnosis and effective control of parasitic infections.

#### Further research

Additional studies are needed to investigate anthelmintic resistance and to assess the economic impact of parasitic infections in equine production systems.

## Discussion

The presence of the various species identified could be linked to a combination of environmental factors and management practices that favour their persistence. Our results confirm several observations made by Mesafint et al.^2^

In Ethiopia, forty-three species of helminth parasites were identified in equids, highlighting the considerable diversity of gastrointestinal parasites affecting horses and related animals in tropical environments, including Strongylus, Cyathostomum, Strongyloides, Anoplocephala, Gasterophilus, Dictyocaulus, Oxyuris, Habronema, Parascaris, Cryptosporidium parvum, Eimeria leuckarti, and Giardia species.

However, the differences observed in the diversity of parasites can be explained by climatic factors, different management practices, or different diagnostic methods and samples.

The high prevalence observed (88.80%) could also be attributed to ineffective deworming, possible anthelmintic resistance or inadequate management practices. Our results are comparable to those of Badji et al.^4^ in Senegal, Ola-Fadunsin et al.^1^ in Nigeria and Yahaya et al.^6^, who reported a dominance of strongyles, followed by *Parascaris equorum*.

The geographical disparities observed in Abidjan District suggest a significant influence of local ecological conditions and practices on parasite dynamics. These observations corroborate the findings of Mourad, (2018) in Algeria, where similar regional variations were observed.

Our results also show that the type of structure (riding school or breeding farm) influences the prevalence of certain parasitic species. Horses in riding schools are more exposed to *Strongylus* sp., *Anoplocephala*, *Fasciola hepatica* and *Cryptosporidium parvum*, whereas those in breeding farms are more exposed to *Parascaris equorum* and *Gastérophilus spp*. These differences can be explained by the management practices (density, cleaning, feeding, and deworming) specific to each type of structure. These findings are in line with those of Yahaya et al.^6^ in Nigeria and Oli & Subedi^5^ in Nepal.

The multivariate logistic regression analysis further confirmed the significant influence of the type of structure on the occurrence of gastrointestinal parasitic infections in horses. Horses raised in breeding farms showed significantly lower odds of infection with Strongylus sp., Trichostrongyles, Anoplocephala spp., Fasciola hepatica, and Cryptosporidium parvum compared with horses kept in riding schools. These findings may reflect differences in management systems, hygiene practices, stocking density, and parasite control strategies between the two types of structures. In breeding farms, horses are generally subjected to more regular health monitoring and deworming practices, which could reduce environmental contamination and parasite transmission.

Similar observations were reported by Ola-Fadunsin et al.^1^ in Nigeria, who highlighted that management conditions and husbandry systems significantly influenced the prevalence of gastrointestinal parasites in horses. Likewise, Yahaya et al.^6^ reported a higher occurrence of equine gastrointestinal parasites in intensively managed horse facilities characterized by high animal concentration and frequent animal movement. In Ethiopia, Mesafint et al.^2^ also emphasized the role of environmental and management-related factors in the persistence and transmission of equine helminths.

The significantly lower odds observed for Fasciola hepatica and Cryptosporidium parvum in breeding farms may additionally be associated with improved water management and reduced exposure to contaminated environments. According to Mathewos et al.^3^, poor hygiene conditions and inadequate stable sanitation are major factors favoring the dissemination of gastrointestinal parasites in equids. Therefore, the present findings underline the importance of implementing effective hygiene measures, regular parasitological monitoring, and targeted deworming programs to reduce the burden of parasitic infections in horses in the Abidjan district.

## Study limitations

This study has several limitations that should be considered when interpreting the findings. First, the cross-sectional design does not allow for establishing causal relationships between risk factors and parasitic infections, but only identifies associations at a specific point in time. Second, the study was conducted in a limited number of facilities (19 sites) within the Abidjan district, which may restrict the generalizability of the results to other regions with different ecological and management conditions.

Third, although multiple coprological techniques were used to improve diagnostic sensitivity, some parasites with low egg shedding or intermittent excretion may have been underestimated. Additionally, the reliance on fecal examination does not allow for the detection of all life stages of parasites.

Fourth, certain management-related variables (e.g., deworming frequency, feeding practices, and hygiene conditions) were based on information provided by horse owners or managers, which may introduce recall or reporting bias.

Finally, the study did not assess anthelmintic resistance or quantify parasite load (e.g., eggs per gram), which could provide a more comprehensive understanding of infection intensity and control challenges.

## Conclusion

This study revealed a very high prevalence and considerable diversity of gastrointestinal parasites among horses in the district of Abidjan, highlighting a significant equine health concern in Côte d’Ivoire. Fifteen gastrointestinal parasite taxa were identified, including Strongylus spp., cyathostomins (Cyathostominae), Trichostrongylus spp., Parascaris equorum, Anoplocephala spp., Strongyloides westeri, Fasciola hepatica, Cryptosporidium parvum, Oxyuris equi, Gasterophilus spp., Dicrocoelium lanceatum, Eimeria solipedum, Giardia spp., Habronema spp., and Dictyocaulus arnfieldi. The predominance of nematodes, particularly Strongylus spp. and Parascaris equorum, reflects substantial environmental contamination and continuous exposure of horses to infective parasitic stages.

The present study also demonstrated that management-related factors, especially the type of structure, significantly influenced the occurrence of several gastrointestinal parasitic infections. Horses raised in riding schools were more exposed to certain parasites compared with those maintained in breeding farms. Logistic regression analysis showed that breeding farms had a protective effect against infections caused by Strongylus spp., Trichostrongyles, Anoplocephala spp., Fasciola hepatica, and Cryptosporidium parvum. These findings suggest that hygiene conditions, animal density, deworming practices, and overall management systems play an important role in parasite transmission dynamics.

Gastrointestinal parasitoses may considerably affect horse health, welfare, physical performance, and productivity, leading to important economic losses for horse owners and breeders. Therefore, the findings of this study emphasize the urgent need to strengthen integrated parasite control strategies in the Abidjan district. Such strategies should include regular parasitological surveillance, improved hygiene and stable management practices, rational use of anthelmintics to limit the emergence of resistance, and targeted deworming programs based on epidemiological evidence. Strengthening veterinary monitoring and increasing awareness among horse owners, breeders, and equestrian facility managers could substantially contribute to improving equine health and productivity in Côte d’Ivoire.

## Data Availability

Data are available upon request from the corresponding author.

## Abbreviations

ISSMV: Institut Supérieur des Sciences et de Médecine Vétérinaire de Dalaba
CI: Confidence interval
OR: Odds ratio
